# Feasibility of a Mobile Phone–Based Data Service for Functional Insulin Treatment of Type 1 Diabetes Mellitus Patients

**DOI:** 10.2196/jmir.9.5.e36

**Published:** 2007-12-31

**Authors:** Alexander Kollmann, Michaela Riedl, Peter Kastner, Guenter Schreier, Bernhard Ludvik

**Affiliations:** ^2^Department of Internal Medicine IIIDivision of Endocrinology and MetabolismMedical University of ViennaViennaAustria; ^1^Department of eHealth SystemsAustrian Research Centers GmbH - ARCGrazAustria

**Keywords:** Telemedicine, diabetes mellitus type 1, blood glucose self-monitoring, mobile phone, Internet

## Abstract

**Background:**

Patients with type 1 diabetes mellitus (DM1) have to be active participants in their treatment because they are inevitably responsible for their own day-to-day-care. Availability of mobile Internet access is advancing rapidly and mobile phones are now widely available at low cost. Thus, mobile phones have the potential to assist in daily diabetes management and to enable a telemedical interaction between patients and health care professionals.

**Objective:**

The aim of the study was to evaluate the feasibility and user acceptance of a mobile phone–based data service to assist DM1 patients on intensive insulin treatment.

**Methods:**

A software application called Diab-Memory (based on Java 2 Mobile Edition) has been developed to support patients when entering diabetes-related data with synchronization to the remote database at the monitoring center. The data were then processed to generate statistics and trends, which were provided for the patient and his/her health care professional via a Web portal. The system has been evaluated in the course of a clinical before-after pilot trial. Outcome measures focused on patients’ adherence to the therapy, availability of the monitoring system, and the effects on metabolic status. General user acceptance of the system was evaluated using a questionnaire.

**Results:**

Ten patients (four female) with DM1 participated in the trial. Mean age was 36.6 years (± 11.0 years) and prestudy glycated hemoglobin (HbA_1c_) was 7.9% (± 1.1%). A total of 3850 log-ins were registered during the 3 months of the study. The total number of received datasets was 13003, which equates to an average of 14 transmitted parameters per patient per day. The service was well accepted by the patients (no dropouts), and data transmission via mobile phone was successful on the first attempt in 96.5% of cases. Upon completion of the study, a statistically significant improvement in metabolic control was observed (HbA_1c_: prestudy 7.9% ± 1.1% versus poststudy 7.5% ± 0.9%;*P*= .02). While there was a slight decrease in average blood glucose level (prestudy 141.8 mg/dL ± 22.5 mg/dL vs poststudy 141.2 mg/dL ± 23.1 mg/dL;*P*= .69), the difference was not statistically significant.

**Conclusion:**

The results of the clinical pilot trial indicate that this proposed diabetes management system was well accepted by the patients and practical for daily usage. Thus, using the mobile phone as patient terminal seems to provide a ubiquitous, easy-to-use, and cost efficient solution for patient-centered data acquisition in the management of DM1. To confirm the promising results of the pilot trial further research has to be done to study long-term effects on glycemic control and cost-effectiveness.

## Introduction

Self management of type 1 (insulin-dependent) diabetes mellitus (DM1) is essential to prevent acute and long-term complications [1]. Therefore, patients are trained in functional (intensive) insulin therapy (FIT) to independently control their blood glucose levels by multiple daily insulin injections [2].

To monitor important glycemic parameters and detect abnormalities at the earliest possible stage, patients are asked to carefully monitor their blood glucose levels and insulin doses. This information is used to help patients adjust their diabetes regimen.

Currently, paper-based diaries, PC programs [3], or Internet-based data services [4,5] are common. These diaries or services are not always available when needed, resulting in incomplete data and inaccurate data representations and feedback. This fact is often associated with poor compliance with therapy, suboptimal or poor glycemic control, and an increased risk of severe hypoglycemia with potentially serious consequences [6].

**Figure 1:**
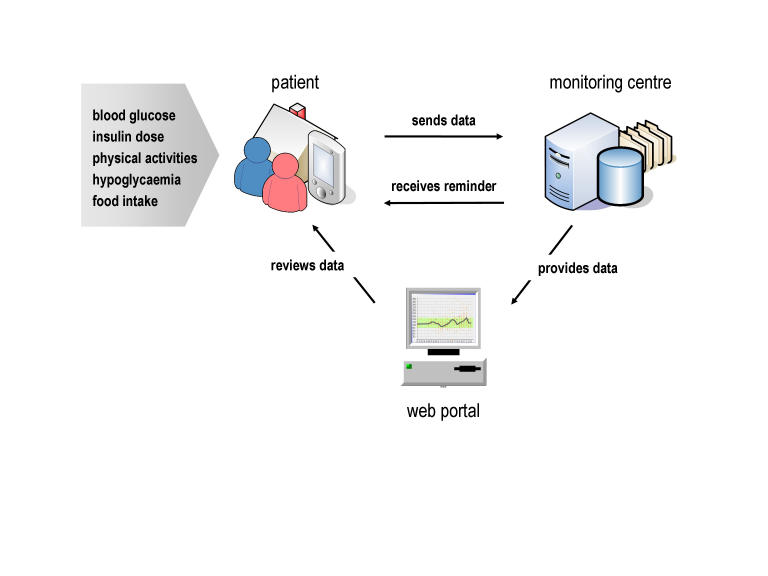
Overview of the mobile phone–based diabetes self-management system

Nowadays, mobile phones and wireless Internet technology are advancing rapidly and are ubiquitously available at low cost [7]. Hence, mobile phones are poised to serve as the universal patient terminal in telemedicine scenarios and data services in the self-management of DM1 [8]. The basic idea presented in this paper is to provide the patient with an easy-to-use mobile phone–based diabetes diary, at low cost, to collect and to transmit daily key measurements to a remote monitoring center (Figure 1). Subsequently, the collected data are processed automatically, resulting in statistics and graphical data that are accessible to the patient via a Web portal.

Several mobile phone–based approaches to support DM1 patients in their daily self- management have already been pursued using Short Message Service (SMS) text messaging [9-15], wireless Internet (WAP) [16,17], or Bluetooth functionality [18,19] of mobile phones. Mobile phones can be used for standard voice communication and transmission of a variety of multimedia information (text, audio, images, video), thus making them the patient terminal of choice for interactive communication and information exchange [20]. Data can be entered using the numeric keypad, or mobile phones can serve as a hub to enable wireless [18,19] or wired [21,22] data transfer from measurement devices (eg, blood glucose meter). The data are then forwarded to a central database via mobile Internet or text messaging.

The mobile phone can also provide an additional link between the health care professional and the patient for personalized feedback (eg, reminders, statistics, or medical advices) [9,10,14,16], education [12,13], or motivation [23]. In addition to technical feasibility, several studies have already pointed out the impact on adherence to therapy [11,12] and self-management [10] when mobile phones are used. Reports of improved glycemic control are diverse. While some studies reported decreased glycated hemoglobin (HbA_1c_) levels [12,15,16], others found no significant effects [11,17].

Differing results regarding improved glycemic control were also reported by large-scale review studies on telemedical intervention in diabetes management [24,25]. Hence, the authors recommended more research to study the impact of technical innovations on improved disease self-management, medical outcome, and cost-effectiveness.

The aim of the present study was to determine the feasibility and level of acceptance of a low-cost, mobile phone–based data service to support DM1 patients treated with FIT.

## Methods

The mobile phone–based, patient-centered diabetes management system was built using state-of-the-art Internet technology and comprised the following:

1. *Patient terminal*: The mobile phone served as a patient terminal to support the patients in recording self-measurements, to trigger data transmission to the monitoring center, and to receive feedback via text messaging.
2. *Monitoring center*: A 24-hour accessible server system received, stored, and processed the data. Central components were user and data management to ensure security, integrity, and traceability of data. Role-based, hierarchical user management guaranteed that only authorized users were able to view, edit, or enter data.
3. *Graphical data representations and reminder*: An automated process analyzed incoming values in order to generate statistics, trends, and graphical representations of the data. In response to the incoming data, reminder messages were generated and sent to patients’ mobile phones using text messaging.
4. *Web portal*: Data were accessible by patients and health care professionals using a standard Web browser.

### Mobile Phone–Based Patient Terminal

**Figure 2:**
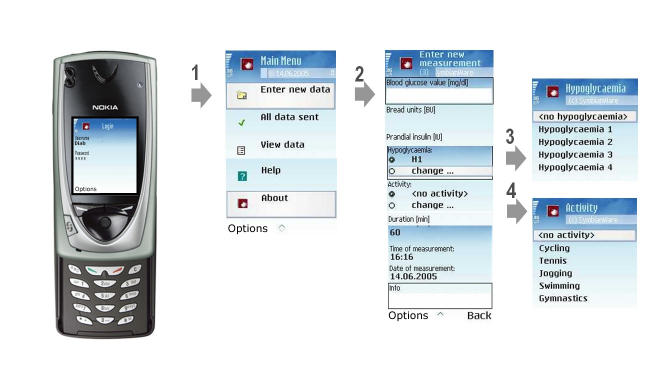
Diab-Memory interface on the mobile phone

A software application called Diab-Memory, based on Java 2 Mobile Edition (J2ME), was developed to support the patients in entering diabetes-related data: (1) blood glucose level, (2) injected insulin doses, (3) content of carbohydrates in meals, (4) well-being, and (5) physical activities. Data were remotely synchronized to the database at the central monitoring center. The graphical user interface is shown in Figure 2.

After logging in, the patient was guided through the data acquisition process. The data were entered manually using the alphanumeric keypad of the mobile phone. The Diab-Memory software application provided an appealing user interface by using metaphoric elements.  The patient was guided through the data acquisition process: (1) user authentication, (2) data entry (eg, blood glucose, content of carbohydrates in meals, insulin dose), (3) hypoglycemia classification if appropriate (1 to 4), and (4) physical activity if performed (eg, tennis, jogging).

To decrease error rate, entered values were immediately checked for plausibility. In case of values above or below predefined thresholds, the patient was warned and asked to enter data again before the data were stored into a database on the mobile phone. The patient could transmit the values immediately or initialize the synchronization process at a later time via mobile Internet-based data transfer using the General Packet Radio Service (GPRS).

### Web Portal

**Figure 3:**
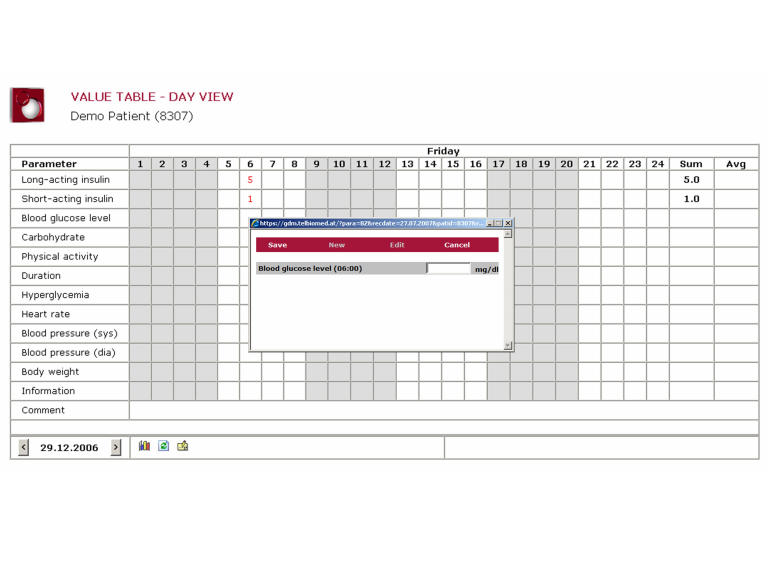
The diabetes data template

The Web portal allows authorized users to access the data via a PC and Internet browser. The graphical user interface components provided the user with a quick overview and supported straightforward navigation. Transmitted values could be accessed easily by the diabetes data template (Figure 3). The patients were also able to enter data using this template. By clicking on the appropriate box in the matrix (hour x, parameter y), a data input window appeared in which the relevant parameter could be entered. Data editing was also possible in the same way.

**Figure 4:**
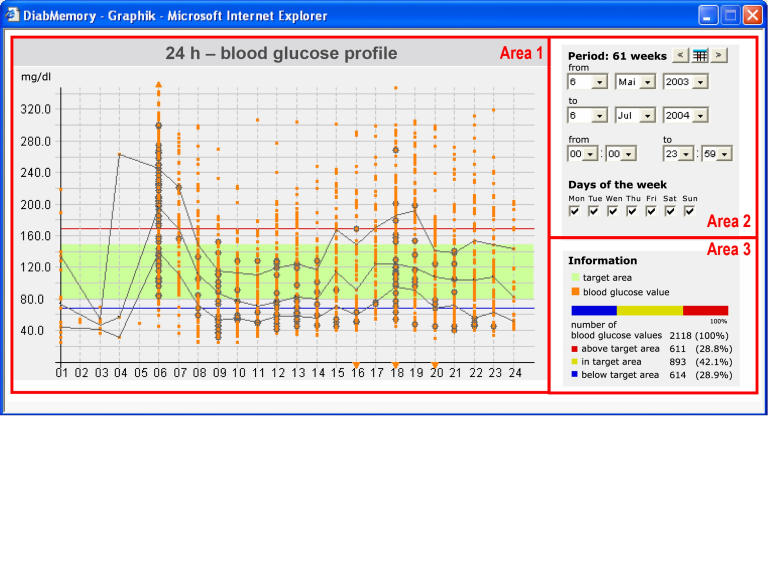
Graphical representation of patient blood glucose data

Statistics and graphical representations of the transmitted data were accessible via Web portal using a JAVA applet (Figure 4). The applet was executed in a pop-up window. First, all available datasets corresponding to a given patient were automatically downloaded to the browser. Thereafter, the user was able to quickly navigate through the datasets without the need for further data download.

A typical 24-hour blood glucose profile is shown in area 1 of Figure 4. The green band indicates the target blood glucose level (80 mg/dL to 150 mg/dL). In addition, the median, upper/lower quartile, and individual upper (red line) and lower (blue line) alert levels are displayed. Transmitted blood glucose measurements were assigned to the corresponding time of day (hour 1-24) and are shown as orange spots. The user was able to navigate through the data by using time navigation (area 2). A summary of the presented data is given as a bar chart in area 3. The blue bar indicates the number of blood glucose values below the target level, and the red bar indicates the number of blood glucose values above the target level. The number of blood glucose values within the target range is represented by the yellow bar.

### Clinical Evaluation

The developed diabetes management system was evaluated in the course of a clinical trial that had been approved by the ethics committee of the Medical University of Vienna (EK 485/03, January 13, 2003). Inclusion criteria were diagnosis of DM1, poor adherence to FIT resulting in suboptimal or poor glycemic control with a lower HbA_1c_value > 7.5%, age 18-80 years, and the ability to operate a mobile phone. Patients with implanted pacemakers or cardioverter defibrillators were excluded from the study.

Ten adults with DM1 agreed to participate in the trial. After signing the informed consent document, the patients were registered to the service and received a secure username/password combination to access the service via the mobile phone or Web portal. The patients were equipped with a mobile phone (Nokia 7650, Nokia, Finland) with the Diab-Memory software application pre-installed and a step-by-step manual.

The patients were instructed to track daily blood glucose measurements and to register the following using the mobile phone: injected basal and bolus insulin doses, content of carbohydrates in meals (1 carbohydrate [bread] unit equals 12 g of glucose), physical activities, and symptoms of hypoglycemia. In cases of less than three successful data transmissions per day, an automatic reminder message was sent to the patient’s phone via SMS. Although the physician could assess the data via Web portal, medical intervention was not planned. During the study, a help desk was established at the monitoring center. Skilled personnel handled questions from users and were responsible for training.

At the end of the 3-month study period, the patients were reviewed in the diabetes clinic and their HbA_1c_was checked. To assess patient satisfaction with the system, the patients were asked to fill in a questionnaire.

### Data Analysis and Statistical Methods

Primary outcome measures were patients’ adherence to FIT, overall availability of the monitoring system, general user acceptance, and usability. We expected that more than 75% of prescheduled measurements would be performed and transmitted to the monitoring center, less than 25% of participants would drop out due to any reason, and the availability of the system would be at least 95%.

Baseline statistics and frequencies of data transmissions and the received data were calculated by standard statistical methods using R statistical software, version 2.4.1 (R Foundation for Statistical Computing, Vienna, Austria) [26]. To assess usability of and patient satisfaction with the system, the cumulative monitoring period, the cumulative transfer sessions, and the total number of received parameters were calculated.

Cumulative monitoring periodis the sum of the number of days from the first to the last successful data transmission for all participants. Cumulative transfer sessions indicate the overall number of datasets transmitted successfully to the monitoring center. Cumulative received parameters add up the number of individual parameter values as entered, transmitted, and received during the cumulative monitoring period.

The secondary endpoint of the study was the impact on glycemic control. HbA_1c_values were therefore measured at the beginning and end of the study period. Additionally, averaged blood glucose levels from the first 14 days of the study period were compared to blood glucose levels of the last 14 days of the study period. The significance of pre- and post-monitoring differences were assessed using the Wilcoxon signed rank test for paired data.

## Results

Table 1 summarizes patient characteristics and monitoring results. Ten patients (four females) with DM1 participated in the trial. Mean age was 36.6 years (± 11.0 years). During the 3 months of the study, a total of 3850 log-ins (3478 via mobile phone and 372 via Web portal) were registered. The total number of received values was 13003 (1300 ± 315 per patient), corresponding to an average of 14 transmitted parameters per patient per day. Data transmission via mobile phone was successful on the first attempt in 96.5% of cases. The availability of the system was 98%.

There were no dropouts during the study period. On 780 out of 920 cumulative monitoring days, at least three blood glucose values were sent, which indicates an adherence rate of 85%. There were 294 (29 ± 23 per patient) SMS reminders sent in the evening. An SMS reminder was sent if less than three blood glucose measurements had been received that day.

**Table 1:** Patient characteristics and monitoring results

	Total	Mean (SD)	Percentage
Total number of patients	10		
Female patients	4		
Dropouts	0		
			
Age, years		36.6(11.0)	
			
Cumulative monitoring period, days	920	92 (0)	100
Cumulative monitoring days where > 3 blood glucose measurements were received	780	78 (18)	84.8
			
Number of SMS reminders	294	29 (23)	
Data corrections via Web interface	183	18 (22)	
			
**Cumulative transfer sessions**	3850		100
Log-in via mobile phone	3478		90.34
Log-in via desktop PC	372		9.66
			
**Cumulative received parameters**	13003	1300 (315)	100
Blood glucose, mg/dL	4294	429 (121)	33.02
Basal insulin dose rate, insulin unit	1414	141 (47)	10.87
Bolus insulin dose rate, insulin unit	3686	369 (120)	28.35
Carbohydrate units, bread unit	3368	337 (83)	25.90
Hypoglycemia	241	24 (25)	1.85

All 10 participants were asked to answer questions (Table 2) at the end of the trial. Seven patients returned completed questionnaires. They reported that they already had experience using a mobile phone and that the Diab-Memory software application was easy to learn and easy to use. They stated that the navigation and the data entry were practical for regular daily use. Problems in reading the display were not reported.

The data acquisition procedure, including blood glucose measurements, log-in, data entry, and data transmission took an average of 3 minutes. Six out of seven patients had already used a hand-written or PC-based diabetes diary. All patients agreed that they found the service helpful. Five out of seven patients responded that an additional teleconsultation with the responsible health care professional at the hospital based on tracked values would be desirable.

**Table 2:** Patient questionnaire (translated from German) on the diabetes management service (n = 7)

Q1	Have you used a mobile phone prior to this study?	yes: 7	no: 0	
Q2	Was the display legible?	yes: 7	no: 0	
Q3	Did you experience problems while inputting data?	yes: 2	no: 5	
Q4	Was the menu prompt easy to navigate?	easy: 5	fair: 2	
Q5	Amount of time required for data entry (on average):	< 2 minutes: 3	> 2 minutes: 4	
Q6	Have you used mobile Internet services for mobile phones prior to this study?	yes: 3	no: 4	
Q7	Length of your training period:	one day: 6	several days: 1	
Q8	Did you send data immediately after measurement?	yes: 2	no: 5	
Q9	Before this study, did you record diabetes-related data at regular intervals?	yes: 6	no: 1	
Q10	Did you use a diary to record your data?	paper-based: 4	electronic: 2	
Q11	How often were your data examined by the responsible physician?	monthly: 1	once every 3 month: 6	
Q12	Length of time spent in doctor’s office per visit:	1-2 hour: 1	2-3 hours: 2	3-4 hours: 4
Q13	Amount of money spent on diabetes-related medication/equipment per month:	€0: 0	< €25: 5	€25-50: 2
Q14	Do you think that the electronic patient diary is a good concept?	very good: 6	good: 1	unusable: 0
Q15	Would you recommend this service to other patients?	yes: 6	no: 1	
Q16	Would you like to continue to use this service?	yes: 4	no: 2	
Q17	Would you use this service even if you have to pay for it?	yes: 4	no: 3	
Q18	If yes, how much would you spend on this service?	< €5: 1	€5-10: 2	
Q19	Do you have Internet access at home?	yes: 6	no: 1	
Q20	Do you find the “up-to-date trend charts” and “statistics” useful?	yes: 5	no: 2	
Q21	Did you inform your colleagues and/or friends about the service?	yes: 5	no: 2	
Q22	Would you like your physician to be more involved?	yes: 5	no: 2	
Q23	Did you discuss this service with your family doctor? His/her first impression was…	positive: 2	sceptic: 0	
Q24	Additional comments			
	I found it very useful to store the data on the mobile phone and to transmit the summarized data once a day.			
	I’m afraid that the doctor-patient relationship will get lost when I’m using this system.			

Regarding the clinical outcome, we found a statistically significant decrease in HbA_1c_(7.9% ± 1.1% vs 7.5% ± 0.9%;*P*= .02) and a slight but not statistically significant decrease in average blood glucose level (141.8 mg/dL ± 22.5 mg/dL vs 141.2 mg/dL ± 23.1 mg/dL;*P*= .69).

When data from the first 2 weeks of the study were compared to data from the last 2 weeks of the study period, the following trends were observed, but all remained not statistically significant: The number of blood glucose measurements above the 150 mg/dL threshold decreased (39.9% to 37.7%;*P*= .07), the number in the normal range decreased (43.1% to 42.0%;*P*= .13), and the number of blood glucose measurements below the 80 mg/dL threshold increased from 17.0% to 20.3% (*P*= .82).

Table 3 summarizes and compares metabolic control during the first and the last 2 weeks of the study period.

**Table 3:** Metabolic control: comparing the first and last 2 weeks of the study period

	Before the Study	After the Study	*P* Value
HbA_1c_, mean (SD)	7.9% (1.1%)	7.5% (0.9%)	.02
	**First2 Weeks of Study**	**Last2 Weeks of Study**	
Blood glucose, mean (SD)	141.8 mg/dL (22.5 mg/dL	141.2 mg/dL (23.1 mg/dL	.69
Number of reported hypoglycemia values, mean (SD)	4 (5.9)	3 (3.9)	
Total number of blood glucose measurements transmitted (%)	725 (100%)	595 (100%)	
Above 150 mg/dL threshold	289 (39.9%)	224 (37.7%)	.07
In normal range (80-150 mg/dL)	313 (43.1%)	250 (42.0%)	.13
Below 80 mg/dL threshold	123 (17.0%)	121 (20.3%)	.82

**Figure 5:**
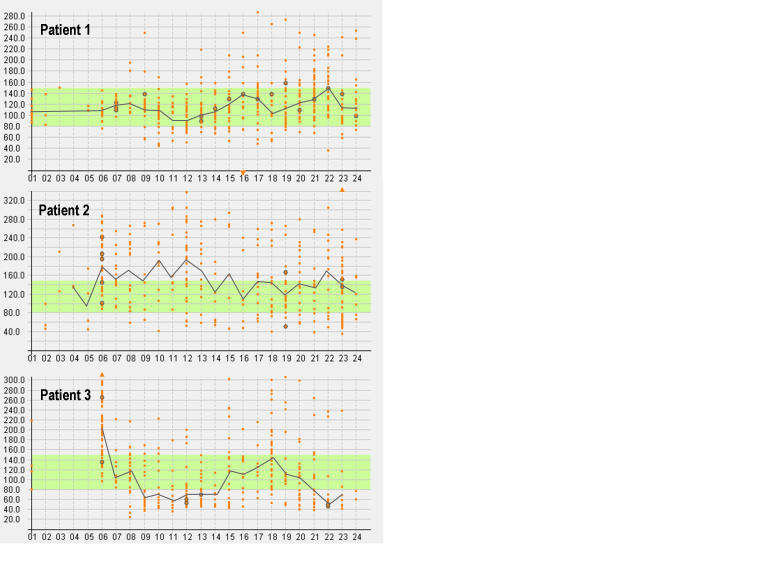
Visualization of the 24-hour blood glucose profiles of three participants

Figure 5 shows the graphical representation of typical 24-hour blood glucose profiles from three patients who participated in the trial. Patient 1 showed a stable condition, in the sense that about 87% of the blood glucose measurements were within the target range of 80 mg/dL to 150 mg/dL. Patient 2 showed increased blood glucose levels in the morning, whereas the blood glucose level in the afternoon was under control. Patient 3 showed poor glycemic control in the morning and the evening. Table 4 adds characteristic values for the same three patients.

**Table 4:** Characteristics of three participants (corresponding 24-hour blood glucose profiles appear in Figure 5)

	Patient 1	Patient 2	Patient 3
Gender	female	male	male
Age (years)	35	30	47
Total number of blood glucose measurements transmitted (%)	469 (100%)	325 (100%)	351 (100%)
Above 150 mg/dL threshold	42 (9%)	127 (39%)	74 (21%)
In normal range (80-150 mg/dL)	408 (87%)	163 (50%)	180 (51%)
Below 80 mg/dL threshold	19 (4%)	35 (11%)	97 (28%)

## Discussion

This paper assessed the technical feasibility and usability of a Web-based data service for DM1 patients using a mobile phone as the patient terminal. The system was evaluated in the course of a clinical pilot study, indicating broad acceptance and significant improvement in metabolic control.

Rapid advancements in information and communication technologies open new possibilities for improved DM1 therapy management. In recent years, several mobile and Web-based telemedicine applications have been developed in order to support diabetes patient self-management. Reviewing the literature, telemedicine services in diabetes care are feasible and acceptable, but evidence of improved glycemic control is weak [24,25,27]. This may be due to poor adherence of patients to the therapy regimen and/or refusal to use telemedicine equipment on a regular basis in day-to-day-care [17]. Because of the ready availability of mobile phones and the possibility of using mobile phones for standard communication issues as well, these devices may serve as patient terminals for health data acquisition in many future telemedicine scenarios.

### Feasibility (Primary Endpoint)

The results of the study have shown that a J2ME-based software application running on widely available off-the-shelf mobile phones provides a robust, easy-to-use, and secure platform for interacting with the diabetes management system. The results of our pilot trial have indicated a high success rate of data transmission (96.5%). In only 3.5% of the cases, a log-in to the service was recognized but data transmission failed, most likely due to a lack of GPRS network connectivity. Since the data are stored in a database on the mobile phone, the transmission could be postponed until network connectivity again became available.

The J2ME software application allows improved usability through the design of user-friendly graphical user interfaces. It uses metaphoric elements and provides data entry plausibility checks. Errors can therefore be excluded at origin, leading to a low error rate. The results show that only 1.4% (183/13003) of cases required correction of the transmitted value via the Web portal (see Table 1). User acceptance was 100%, as indicated by the fact that there were no dropouts and two patients chose to continue using the service even after the study had been closed.

Two participants reported technical problems during the study. One patient complained of a keypad malfunction, and the other reported permanent connection problems due to lack of GPRS network connectivity at home.

Both problems were solved: in the first case, the mobile phone was replaced, and in the second case, the patient was asked to postpone data transmission until entering an area of acceptable network connectivity.

Although the patients also had the option of using a Web portal for data input, over 90% of values were transmitted via mobile phone. The remaining 10% of values were entered via the Web portal mainly by two patients who used the Internet access at their work. User activities via the Web portal were not analyzed in detail as it was not within the scope of the study. The availability of the system was 98%, which means that patient data transfer was almost always possible during the entire study period.

Five out of seven patients rated visualization and up-to-date statistics as very helpful for self-management and monitoring. It was seen as useful to navigate through the data over time in order to make changes in glycemic control visible. This could be convenient in the case of therapy or lifestyle changes to allow early identification of illness patterns. Two participants felt overwhelmed with self-interpretation of the data and stated that it would be more helpful to have direct feedback from the health care professional. In those cases, interactive representation of data can provide a basis for the health care professional to derive medical advice or instructions.

Effective management of chronic disease requires a close partnership between the patient and health care professional, which can be supported by contemporary information and communication technologies. Telemedical data services are not intended to replace patient-physician contact but rather to assist the patient in diabetes self-management. We propose an individual therapy schedule for each patient. Efficient therapy management alternates telemedical interventions and clinic visits depending on the health status and independence level of the patient.

### Individual Metabolic Status (Secondary Endpoint)

Aside from simplification and increased efficiency of documentation, the system was able to improve patients’ adherence to the treatment scheme. We expected that more than 75% of prescheduled measurements (at least three blood glucose measurements daily) would be performed and transmitted to the monitoring center. The results indicated a therapy adherence rate of 85%, and most likely as a consequence of improved adherence, a significant improvement in HbA_1c_and a decreased number of overshoots of the 150 mg/dL blood glucose threshold.

The observed positive effects may have been influenced by the small sample size and the fact that patients where selected neither randomly nor consecutively. We cannot exclude the possibility that subjects who subscribed to the study had an enthusiasm for technical innovations and good therapeutic compliance. The small sample size is also unlikely to represent all patients with DM1, which may influence the robustness of the results.

During the study period, there was no interaction between the health care professional and the patient. Patients who transmitted less than three successful blood glucose measurements were automatically sent a reminder message to their mobile phone. This feature may also have contributed to improved quality of self-managed therapy.

### Operating the Monitoring Center

Because of the limited number of users and expected workload, neither specific hardware components nor particular server architecture were considered. In order to guarantee almost 100% availability of the monitoring center, hosting issues and maintenance of technical equipment and software had to be addressed.

Additionally, a help desk was established during the study. The help desk was accessible via telephone or email. Skilled personnel handled questions and were responsible for training subjects after registration. Initial training took place by telephone and took approximately 20 minutes. Participants were introduced to the use of the software on the mobile phone and the Web portal. Patients were then asked to explore the functionality of the system independently. Data from the initial day were therefore not used for further analyses. Six out of seven patients were able to operate the service after one day; only one participant needed additional training. Hence, frequency of help desk assistance for technical issues was low.

### Costs

Patients incurred no costs during the pilot trial. The mobile phone was provided with a free data bundle and the Diab-Memory service. There were also no clinic costs as the health care professional was not involved in therapy management during the study. For further large-scale telemonitoring scenarios, costs to patient, service, and clinic have to be considered.

As network providers offer a wide range of packages and fees, including or excluding data bundles, costs to the patients are difficult to estimate. However, assuming that the patient already owns a mobile phone and the contract includes a data bundle, an extension would not be necessary because the amount of data for Diab-Memory is marginal. If a data bundle is not included, the required upgrade would cost about €10 per month.

The costs of the service and the health care professional are also difficult to assess. The operating costs depend on the number of users and complexity of the service, while costs for the health care professional are dependent on time spent on monitoring. To reduce labor costs, automatic data analysis and alerts for the physician would be helpful in case of detected abnormalities.

Aside from improved medical care through patient empowerment, it is predicted that home monitoring would be cost-effective. The idea of home monitoring is to guide the patient to the best possible health outcomes and to identify problems at the earliest possible stage. Hence, long-term complications and emergencies could be avoided, resulting in reduced costs from clinic visits. This may free financial resources of clinics. However, these processes are very complex, involve many stakeholders (and financial investors), and differ between health care systems. To study pecuniary effects of telemedical data services in DM1, further research and large-scale studies are needed.

### Future Developments

The promising results from the present pilot study will lead to further innovations to improve the diabetes management system in near future. These are related to data acquisition, automatic feedback and alerts, and communication between patient and health care professional.

#### Data Acquisition

Although the Diab-Memory software was well accepted, an automated method to assess the measurements would be helpful. Running J2ME-based software applications on mobile phones will support access to integrated mobile phone features like Bluetooth and Infrared technology. This technology could be used to transfer data from the glucose meter to the mobile phone automatically. Several studies have already highlighted the technical feasibility of blood glucose meters supporting automatic data transfer. However, for effective diabetes management, further relevant data, including insulin doses and physical activity, are required. At the moment, these data have to be added manually using a numeric keypad. A promising solution could be the use of near field communication (NFC) in telemonitoring scenarios. Near field communication supports a touch-based method for data acquisition using the mobile phone [28], and, in addition to allowing the capture of readings from a meter, it provides access to data stored on radio frequency identification (RFID) tags (eg, electronic barcodes), which could be embedded in future diabetes care monitoring scenarios.

#### Automatic Feedback and Alerts

The present study showed the feasibility of reminder messages sent via SMS to the patient. Future studies should evaluate whether close monitoring of therapy parameters combined with automatically generated feedback and instructions can further improve the metabolic control of patients with DM1. To facilitate this, automated analysis and rule-based interpretation of transmitted data on an individual basis would be helpful.

#### Communication

The study revealed the need for teleconsultation between the patient and the health care professional regarding the blood glucose data provided. One patient stated that he would be afraid to lose personal contact with the physician if using this system. Hence, the Web portal must provide a collaboration and communication platform between the patients and their health care professionals in order to allow an additional link between these partners.

### Conclusion

The prototype was designed to demonstrate feasibility, to evaluate user needs, and to help us understand the complex processes of DM1 self-management. The results of the present study indicate that diabetes management based on mobile phones and the Internet is technically valid and well accepted by patients for daily use and resulted in improved glycemic control. Thus, using mobile phones as patient terminals seems to provide a ubiquitous and easy-to-use solution for patient-centered data acquisition in the management of DM1. The results and the recommendations for improvement provide the basis for further research. To confirm the promising results of the pilot trial, further research must be done to study long-term effects on glycemic control and cost-effectiveness. In the next step, a randomized trial must be carried out with a larger sample size and physician involvement.

## Abbreviations

DM1: type 1 diabetes mellitus
FIT: functional insulin therapy
GPRS: General Packet Radio Service
HbA1c: glycated hemoglobin
J2ME: Java 2 Mobile Edition
PC: personal computer
SMS: Short Message Service
